# Education and Training on Infection Prevention and Control Provided by Long-Term Care Homes to Visitors: A Scoping Review

**DOI:** 10.3390/nursrep15010017

**Published:** 2025-01-10

**Authors:** Rachel MacLean, Pamela Durepos, Lisa Keeping-Burke, Richelle Witherspoon, Patricia Morris, Caroline Gibbons, Natasha Taylor, Rose McCloskey

**Affiliations:** 1 Department of Interdisciplinary Studies, University of New Brunswick, 3 Bailey Drive, Fredericton, NB E3B 5A3, Canada; 2 Faculty of Nursing, University of New Brunswick, 33 Dineen Drive, Fredericton, NB E3B 3X9, Canada; p.morris@unb.ca; 3 Department of Nursing and Health Sciences, University of New Brunswick, 100 Tucker Park Rd., Saint John, NB E2K 5E2, Canada; lkeeping@unb.ca (L.K.-B.); rmcclosk@unb.ca (R.M.); 4 JBI Centre of Excellence, The University of New Brunswick (UNB) Saint John Collaboration for Evidence-Informed Healthcare, 100 Tucker Park Rd., Saint John, NB E2K 5E2, Canada; r.witherspoon@unb.ca; 5 Library Services, University of New Brunswick, 5 Macaulay Ln, Fredericton, NB E3B 5H5, Canada; 6 School of Nursing, Université de Moncton, 18 Avenue Antonine-Maillet, Moncton, NB E1A 3E9, Canada; caroline.gibbons@umoncton.ca; 7 Department of Psychology, University of Regina, 3737 Wascana Pkwy, Regina, SK S4S 0A2, Canada; natasha.taylor@unb.ca

**Keywords:** family, infection prevention and control, long-term care, nursing, visitors

## Abstract

**Objective:** The objective of this study is to identify, examine, and map the literature on infection prevention and control (IPAC) education and training for visitors to long-term care (LTC) homes. **Introduction:** Visitor restrictions during infectious outbreaks in LTC homes aim to reduce virus transmission to vulnerable residents. The COVID-19 pandemic highlighted the negative impacts of such restrictions, prompting the need for IPAC education for visitors. **Inclusion Criteria:** This review includes research, narrative papers, and grey literature on IPAC education and training for LTC visitors. It focuses on intentional education aimed at preventing infection transmission. Studies not involving visitors or offered in other settings were excluded. **Methods:** Following the JBI methodology for scoping reviews, bibliographic databases (CINAHL, Embase, AgeLine, Medline, and ERIC) were searched from 1990 to present in English or French. Data were extracted by two reviewers, focusing on the educational content, delivery mode, frequency, timing, and qualifications of educators. A narrative summary and descriptive statistics were produced. **Results:** The 26 included documents contained guidelines, policies, educational resources, and opinion papers. Pre-2020, healthcare workers were responsible for educating visitors. Post-2020, more detailed recommendations emerged on the frequency, content, and delivery methods. Key topics included hand hygiene (92.3%), respiratory hygiene (80.8%), and PPE use (73.1%). **Conclusions:** IPAC education and training for LTC visitors is essential for safe visitation. Future research should evaluate the effectiveness of these educational interventions.

## 1. Introduction

Infection prevention and control (IPAC) education and training are essential for protecting residents in long-term care (LTC) homes. LTC homes house aging, frail individuals with chronic health conditions, making them particularly vulnerable to infections [1], compounded by organizational challenges, such as a limited living space and high staff turnover, further complicating IPAC efforts. Historically, visitor restrictions have been seen as critical during infectious outbreaks to protect residents [2,3,4,5]. This practice, dating back to the 1800s, stemmed from the belief that visitors are a vehicle for infections [2,6]. While visitor restrictions have been effective in reducing the risk of infection, they are associated with negative psychological impacts, such as social isolation and depression [7]. These concerns became especially pronounced during the COVID-19 pandemic, intensifying the debate around visitor bans [8].

During the pandemic, global visitor restrictions imposed in LTC homes negatively impacted residents and families, leading to significant efforts to balance infection control, safety, and quality of life [9]. For instance, research since the pandemic onset has highlighted the severe negative effects of visitor bans, including increased depression, agitation, and cognitive decline among residents [10,11] and family members [10,11,12]. These findings emphasize the importance of adopting a balanced approach that takes both infection control and residents’ quality of life into account.

The devastating effects of the COVID-19 pandemic on LTC globally have led to increased efforts to introduce safer visitation strategies [12]. However, the pandemic response highlighted inconsistent regional policies due to a lack of coordination, with homes often working in isolation and struggling to share information effectively [13]. This fragmentation has made it clear that ensuring safety during times of heightened infection risks requires robust IPAC practices. Research has shown improper hand hygiene is a major contributor to the spread of infections, and LTC visitors have been identified as potential carriers of undetected infections, such as tuberculosis and influenza [14,15].

To mitigate these risks, IPAC education and training for visitors is critical. A Canadian study, for example, found notable gaps in visitors’ ability to follow hand hygiene and personal protective equipment (PPE) protocols [16]. While existing research suggests that repetitive, multi-modal education improves IPAC compliance in LTC healthcare workers [17], little is known about how visitors to LTC are educated and trained for IPAC. This scoping review aims to fill this gap by systematically exploring and mapping the various approaches to IPAC education and training for visitors in LTC homes, with a focus on understanding commonly used methods, content, and delivery strategies.

The relevance of this work to nursing is significant, as nurses are integral to the coordination and delivery of IPAC protocols in LTC settings, ensuring staff and visitors adhere to safety measures. This research directly informs nursing practice by mapping visitor IPAC education and training in LTC, which often falls under the responsibility of nursing staff and is a crucial component in safeguarding vulnerable populations. By addressing visitor IPAC education and training, this paper offers valuable insights that can inform future research, policy development, and practical interventions in LTC settings. It contributes to the literature by focusing on the visitor education aspect, an area that has received limited attention in comparison to staff-focused IPAC education and training. In doing so, the paper helps to bridge an important gap in our understanding of how best to manage infection risks in LTC homes while maintaining a safe and supportive environment for both residents and their families.

### Research Questions

The overarching review question is as follows: What IPAC education and training have been recommended and/or implemented for visitors in long-term care homes? The five review sub-questions are as follows:

1. What IPAC education and training policies and guidelines exist related to visitation in LTC?
2. How is education and training related to IPAC delivered to visitors of LTC residents, including frequency, timing, and mode of delivery?
3. What content is included in the IPAC education and training provided to visitors of LTC residents?
4. What qualifications are required by staff who provide education and training to visitors of LTC residents?
5. How has the education and training provided to visitors evolved over time (i.e., pre-pandemic, and throughout the pandemic)?

## 2. Review Criteria

### 2.1. Participants

This review included IPAC education and training activities and practices for visitors to LTC homes. Visitors to LTC are not a homogenous group—their IPAC education needs and visiting patterns vary, impacting the educational resources, content, and modes of delivery needed. Visitors included unpaid caregivers, essential caregivers, volunteers, care partners, family members, and/or friends who entered LTC for the sole purpose of visiting a resident. Any education and training that involved staff but also included visitors was considered. There were no limitations imposed on the age, gender, or ethnicity of a visitor to LTC.

### 2.2. Concept

The concepts examined in this scoping review included all planned and intentional education and training activities, practices, and/or guidelines used for IPAC with visitors in LTC homes. Education included intentional activities, such as demonstrations, to change knowledge, attitudes, or awareness of IPAC practice in LTC. Training included intentional activities to learn IPAC skills or behavior. Education and/or training included but were not limited to in-person, independent, virtual, individual, or group activities. There were no limits regarding the frequency, duration, setting, or provider of the education and training. Any IPAC education and training provided exclusively to staff was excluded.

### 2.3. Context

LTC homes include any setting, such as a nursing home, residential aged care facility, or skilled-nursing homes, that provide health and social services and residential accommodation to people who cannot care for themselves at home. Other settings, such as home or hospital, were not considered as they are outside the scope of this review focused on LTC facilities.

### 2.4. Types of Sources

All variations of mixed-methods, quantitative, and qualitative study designs were considered for inclusion in this scoping review. Any text, policy, opinion, and guidelines meeting the inclusion criteria were considered.

## 3. Methods

This review is registered with Open Science Framework: https://doi.org/10.17605/OSF.IO/NMXUB (accessed on 31 May 2024). This review adhered to the PRISMA-ScR reporting guidelines [18] (Supplementary Material Table S2), followed an a priori protocol [19], and utilized the JBI scoping review methodology [20] including five phases: (i) identifying the research questions; (ii) searching for evidence; (iii) selecting documents; (iv) extracting data; and (v) reporting findings.

### 3.1. Search Strategy

The search aimed to identify both published and unpublished literature. A JBI-trained librarian (RW) developed the search strategy, which was reviewed by the team. The strategy was adjusted for each database (CINAHL, ERIC, AgeLine, and MEDLINE) (Appendix A) to ensure comprehensive results while avoiding irrelevant sources. Education-related terms were initially tested but excluded to broaden the search. Results were limited to French and English language as those are the languages spoken by the research team members. Results were limited from 1990, reflecting the earliest published IPAC guidelines [21] to early 2023, reflecting the early post-COVID period. Reference lists of selected studies were also reviewed for additional.

### 3.2. Information Sources

The databases searched included CINAHL, Embase, ERIC, MEDLINE, and AgeLine as these are large health-, nursing-, and aging-related databases (see Appendix A). Unpublished studies were sought through Google, which identified 89 relevant aging-related websites (Appendix B). Search terms from the database search strategies were used across websites by RM.

### 3.3. Study Selection

**Figure 1:**
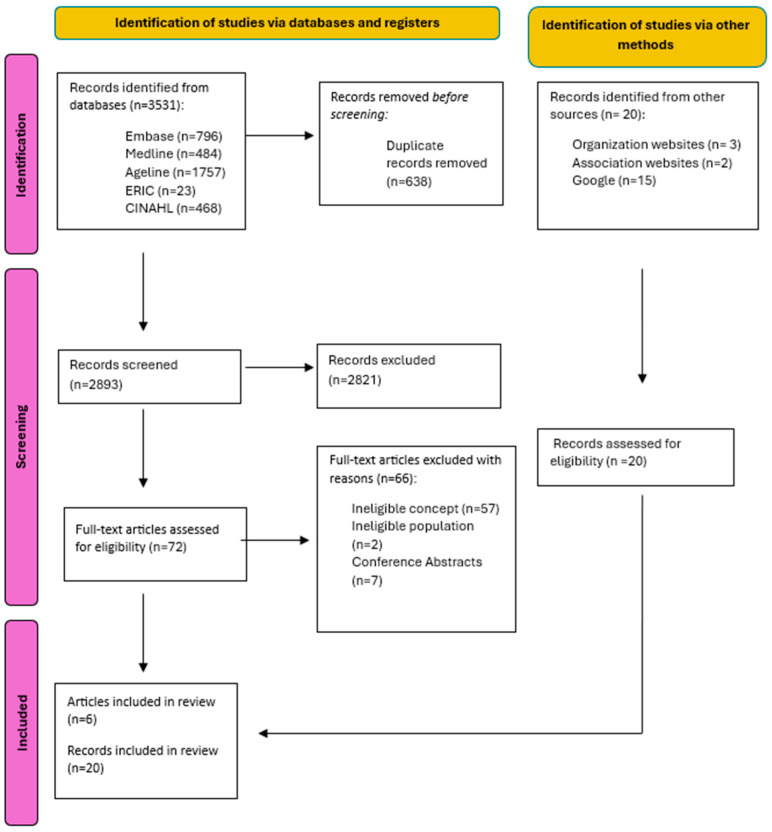
PRISMA flow diagram. Note: Adapted from Page et al. [22].

All records were managed in Covidence (Ventas Health Innovation, Melbourne, Australia) systematic review software. As a pilot test of the screening, the authors screened 50 records together and independently screened 200 abstracts (approximately 5% of identified records) to ensure consistency among reviewers (RM, PD, RMM, LKB, CG, and NT). Titles and abstracts were screened by two independent reviewers with any disagreements resolved by a third reviewer. Next, the full texts of each included document were screened by two independent reviewers. Disagreements between reviewers at this stage were resolved by a third reviewer by reading the full text of the document to decide if it should be included. Excluded studies are listed in the Supplementary Material and a PRISMA flow diagram [22] (Figure 1) was created to visualize the search and screening process.

### 3.4. Data Extraction

A pilot of the data extraction tool (Supplementary Material Table S1) was conducted to ensure consistency. Two reviewers independently extracted data, including content, frequency, and delivery methods, and discrepancies were resolved by a third reviewer. Data from grey literature were extracted into an Excel spreadsheet by RM and PD.

### 3.5. Data Analysis and Presentation

Extracted data were summarized narratively and presented in tables and graphs. The tables display details of included documents, and graphs highlight trends in the findings related to the research questions supported by narrative summaries.

## 4. Results

### 4.1. Study Inclusion

From a total of 3531 identified records, 638 duplicates were removed, 2824 records were excluded through the screening process, and 66 records were excluded after a full-text review. An additional 20 documents were identified through grey literature searches, bringing the total to 26 included documents.

### 4.2. Characteristics of Included Documents

A summary of the characteristics of the included documents is in Appendix C. Most included documents were IPAC guidelines (50%) [3,4,5,8,23,24,25,26,27,28,29,30,31], educational resources (15.4%) [32,33,34,35], and policies (11.5%) [36,37,38]. Most documents originated from Canada (53.9%) [1,3,4,5,25,26,27,28,34,37,38,39,40,41], the USA (26.9%) [8,25,30,31,33,37,42], and Australia (7.7%) [23,35]. Eight documents (30.8%) were developed pre-COVID-19 [3,4,5,24,25,26,36,39], and eighteen (69.2%) were developed after the onset of COVID-19 [1,8,23,27,28,29,30,31,32,33,34,35,37,38,40,41,42,43]. The intended audience for most documents was healthcare workers (50%) [3,4,5,8,23,24,26,30,31,34,36,38,39], visitors (23.1%) [31,32,33,34,35,38], and healthcare/LTC organizations (23.1%) [25,27,28,29,37,41].

### 4.3. Review Findings

The main review question was as follows: what IPAC education and training have been recommended and/or implemented for visitors in long-term care homes? To address this, the findings are presented according to each sub-question. Detailed descriptions of the findings can be found in the Supplementary Material.

#### 4.3.1. Sub-Question 1: What IPAC Education and Training Policies and Guidelines Exist Related to Visitation in LTC? 

As depicted in Appendix C, thirteen documents (50%) were IPAC guidelines for specific infectious diseases, including COVID-19 [23,28,30,31], influenza [4], *Clostridioides difficile* (*c. diff*) [3], and healthcare-associated infections [5]. Six documents (23.1%) were general and/or implementation guidelines for IPAC in healthcare facilities, including LTC homes [8,24,25,26,27,29]. Three policy documents (11.5%) addressed preparing for infectious disease outbreaks and visitation [36,37,38]. None of the documents provided a comprehensive overview of IPAC education and training provided to visitors in LTC, including covering all aspects of IPAC training (i.e., provider, frequency, timing, delivery mode, and content).

#### 4.3.2. Sub-Question 2: How Is Education and Training Related to IPAC Delivered to Visitors of LTC Residents, Including Frequency, Timing, and Mode of Delivery?

**Figure 2:**
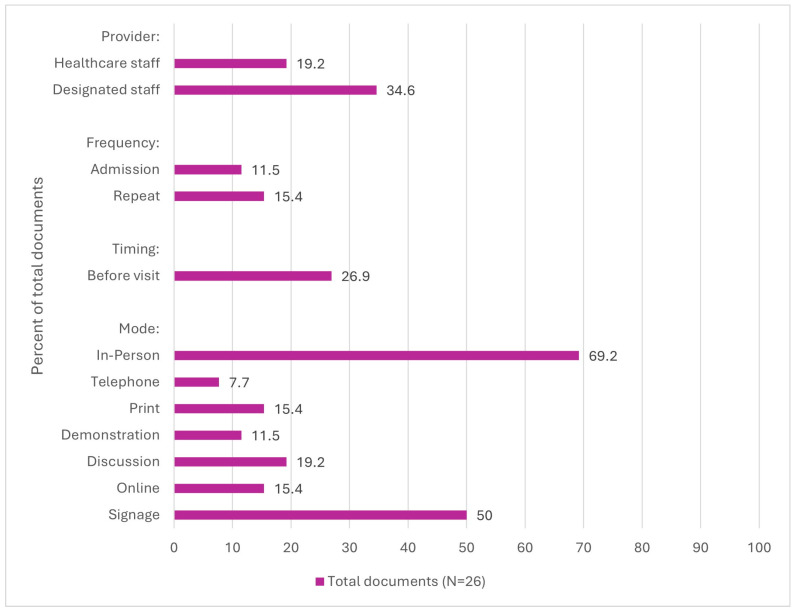
Processes recommended or addressed in documents (N = 26).

**Frequency of Delivery.** As displayed in Figure 2, six documents (23.1%) described the frequency of IPAC education for LTC visitors [8,27,28,31,38,41]. Recommendations included providing education during resident admission and when precautions are implemented [8,28,41], repeating the education [27,31,38,41], and having visitors complete training before their first visit and retrain if non-compliant [38].

**Timing of Delivery.** Seven documents (26.9%) outlined recommendations for the timing of education and training [1,4,23,30,35,38,42]. Recommendations included education and training be provided before visiting a resident, such as upon arrival to an LTC home or when scheduling visitation appointments [1,4,23,30,35,38,42].

**Mode of Delivery.** Twenty-three documents (88.5%) discussed modes of education and training delivery [1,3,4,5,8,23,24,26,27,28,29,30,31,32,33,34,35,36,37,38,39,41,42,43]. Recommendations included in-person delivery (69.2%) [1,3,4,5,8,23,28,29,30,31,33,35,36,37,38,39,41,42,43], signage (50.0%) [4,8,23,26,28,29,30,31,35,36,37,38,42], and discussion/information sessions (19.2%) [1,8,33,35,43]. The CDC emphasized culturally diverse materials tailored to visitors’ language comprehension and education levels [29].

#### 4.3.3. Sub-Question 3: What Content Is Included in the IPAC Education and Training Provided to Visitors of LTC Residents?

**Figure 3:**
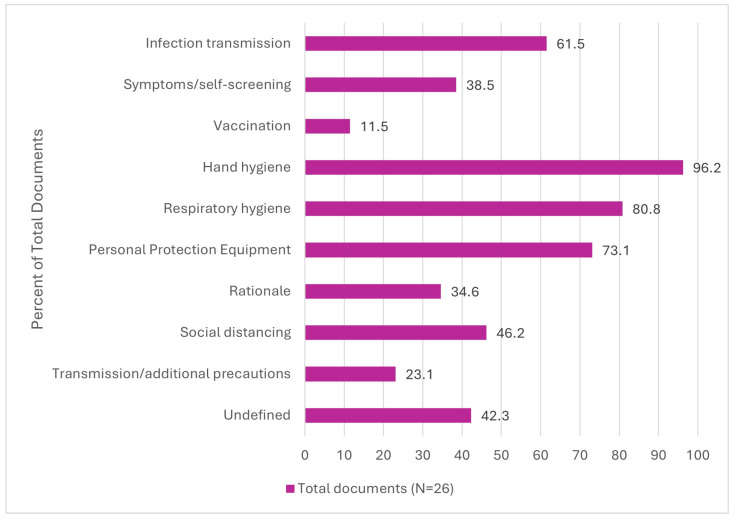
Content recommended or addressed in documents (N = 26).

As outlined in Figure 3, documents included varied recommendations on content to include in the IPAC education and training provided to visitors of LTC residents. The recommended content most frequently included hand hygiene (96.2%) [1,3,4,5,8,23,25,26,27,28,29,30,31,32,33,34,35,37,38,39,40,41,42,43], respiratory hygiene (80.8%) [1,4,5,8,23,25,27,28,29,31,32,33,34,35,36,37,38,40,41,42,43], PPE usage (73.1%) [1,3,4,5,8,23,24,25,26,27,28,30,31,33,34,35,36,37,38,39,40,41,42,43], infection transmission (61.5%) [3,5,25,26,28,29,30,31,32,33,34,35,36,39,40,43], and social distancing (50.0%) [1,23,24,25,26,27,28,30,31,32,34,36,37,38,39,40,41,42]. Eleven (42.3%) documents [8,23,24,25,27,29,36,37,38,41,43] included undefined IPAC content, described as “appropriate” [29,41], “other” [8,36,37], or “specific” IPAC practices [25]. The content recommended the least was vaccination (11.5%) [8,23,30].

#### 4.3.4. Sub-Question 4: Who Provides the Education and Training to Visitors of LTC Residents?

The recommended education and training providers for LTC visitors in the included documents were IPAC professionals and/or designed individuals (42.3%) [8,25,27,28,30,31,33,34,37,40,41], and healthcare workers (19.2%), such as nurses [1,4,5,8,43]. 

#### 4.3.5. Sub-Question 5: How Has the Education and Training Provided to Visitors Evolved over Time (i.e., Pre-Pandemic, and Throughout the Pandemic)?

**Figure 4:**
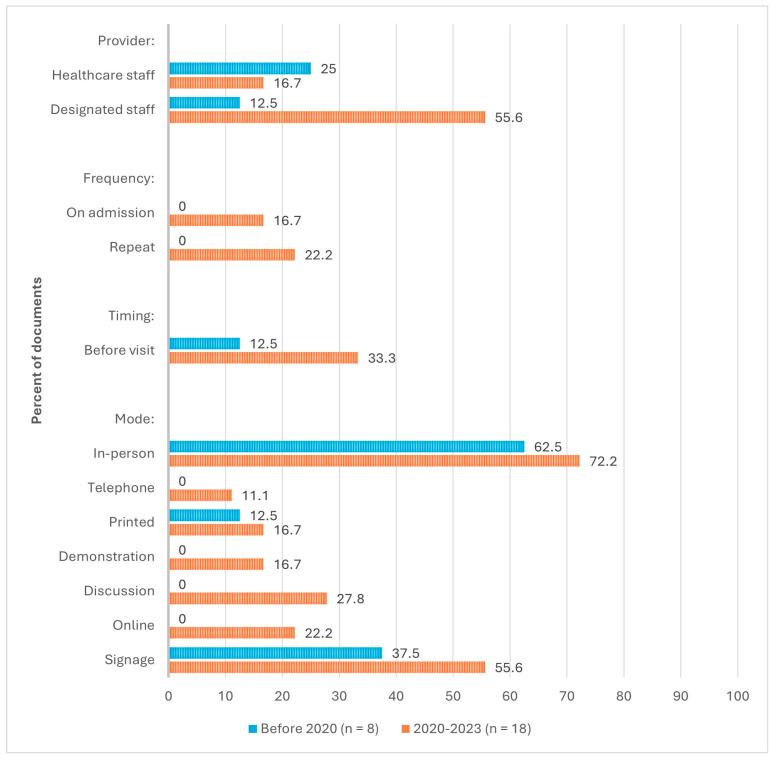
Evolution of recommended processes (N = 26).

**Evolution of Provider, Frequency, Timing, and Mode of Delivery.** As depicted in Figure 4, the recommended provider of IPAC education and training to visitors in LTC was healthcare staff before 2020 (25%) [4,5], and this switched to designated staff (55.6%) [8,25,27,28,30,31,33,34,37,40,41] after 2020. Before 2020, documents did not include recommendations on the frequency and timing of education and training delivery. After 2020, visitor education and training were most frequently recommended to be repeated (22.2%) [27,31,38,41] and provided before a visit occurs (33.3%) [1,23,30,35,36,42]. Before 2020, the recommended education and training delivery modes were in-person (62.5%) [3,4,5,36,39] and through signage (37.5%) [4,26,36]. After 2020, the most frequently recommended modes remained as in-person (72.2%) [1,8,23,27,28,29,33,34,36,37,38,39,41,42,43,44] and through signage (55.6%) [8,23,28,31,35,37,38,42], and recommendations for delivery via telephone, print, demonstrations, discussions, and online modes were introduced. 

**Figure 5:**
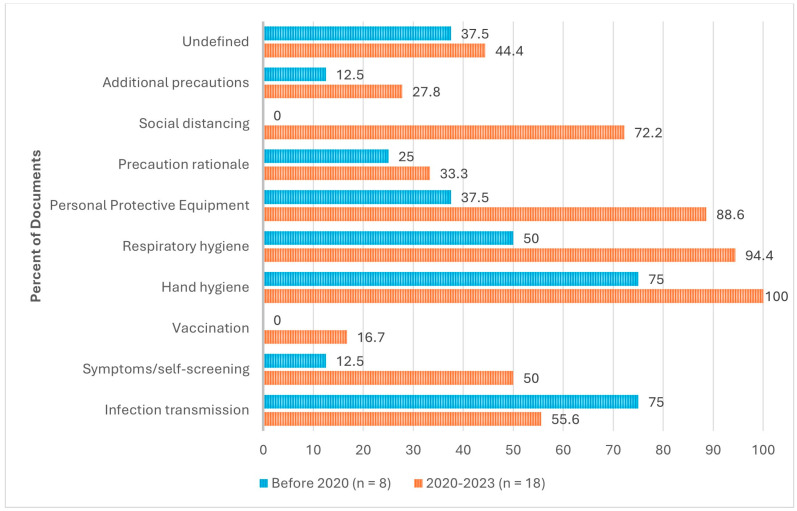
Evolution of recommended content (N = 26).

**Evolution of Content.** As shown in Figure 5, before 2020, the most frequently recommended content for IPAC education and training for LTC visitors was hand hygiene (75.0%) [3,4,5,25,29,39] and infection transmission (75.0%) [3,5,25,26,36,39]. After 2020, the most frequently recommended content was hand hygiene (100%), respiratory hygiene (94.4%) [1,8,23,27,28,29,31,32,33,34,35,37,38,40,41,42,43], and personal protective equipment usage (88.6%) [1,4,5,23,27,28,30,31,33,35,37,40,41,42]. The largest increase in content recommendation was social distancing, from zero documents before 2020 to 72.2% of documents after 2020 [1,23,27,28,31,32,34,37,38,40,41,42].

## 5. Discussion

This paper provides new insights into the evolving landscape of IPAC education for visitors in LTC homes, highlighting both the progress and persistent gaps in education delivery. The findings suggest visitor education was typically recommended at the time of admission, during an infectious outbreak, and prior to visiting. It was predominantly delivered in person, and the core content included hand hygiene, respiratory hygiene, and the use of PPE. However, significant gaps were identified in both the processes and content of IPAC education for visitors.

### 5.1. Changes in Education and Training Provider

Before 2020, there were no standardized recommendations regarding the qualifications of individuals responsible for visitor IPAC education and training in LTC. This review reveals a significant shift post-2020, where the responsibility has been more formally assigned to designated individuals with specialized IPAC training, such as IPAC professionals. This change reflects the increased awareness of the importance of IPAC in preventing the spread of infections, especially during outbreaks, and the growing recognition that care staff may not have the time or expertise to provide this education effectively [10,28]. In some countries, such as Canada, certified IPAC professionals are already recognized as key personnel for overseeing infection control practices [45], but LTC challenges related to staffing and financial constraints often prevent dedicated IPAC professionals from being present in all facilities, potentially leading to gaps in training delivery. Despite this, nurses remain key facilitators of IPAC initiatives in LTC, often bridging the gap between IPAC professionals, care staff, and visitors. Nurses possess clinical expertise and an established rapport with residents and visitors, making them well-positioned to ensure that IPAC education and prevention measures are effectively communicated and adhered to within the facility. They play an integral role in reinforcing IPAC guidelines, monitoring compliance, and addressing challenges in implementing IPAC practices, ultimately contributing to the safety and well-being of vulnerable residents [46].

### 5.2. Evolution of Timing and Frequency of Education

While the importance of repeated visitor education became clear in 2021, partway through the COVID-19 pandemic, this review underscores the need for standardized guidance on the frequency and timing of training and retraining. Evidence suggests that periodic retraining improves adherence to infection control measures [17], but there remains a lack of specificity about the optimal timing for such interventions. Future research should aim to develop best practices for training schedules, assisting LTC homes in balancing the need for effective visitor education with the practical constraints they face in resource allocation.

### 5.3. Delivery Methods

The methods of delivering IPAC education and training evolved significantly after 2020. Pre-2020, in-person training and signage materials were the most common methods. However, post-pandemic documents outlined varied delivery options, including online resources, telephone communication, and one-on-one sessions. This broader range of methods aligns with adult learning theory [47], recognizing that visitors in LTC homes have diverse learning preferences and needs. Offering multiple delivery modes could increase accessibility and engagement, which is especially important for addressing a wide range of literacy levels, language differences, and learning styles among visitors.

### 5.4. Content of IPAC Education

The content of IPAC education and training for visitors primarily focused on essential infection control measures, such as hand hygiene, respiratory hygiene, and PPE usage, aligning with global public health recommendations from organizations like the World Health Organization and CDC [48]. However, a notable gap was identified in the inclusion of vaccination information—less than 12% of the documents reviewed recommended including this crucial content in visitor education and training. This is especially significant given the increasing role of vaccinations in preventing LTC infectious disease outbreaks, such as influenza, which are often associated with high resident mortality rates [4]. Including vaccination education in visitor training could strengthen infection prevention efforts, as vaccinated visitors are less likely to transmit infections [49,50].

Despite the growing evidence supporting the effectiveness of vaccines in reducing infectious diseases, vaccine hesitancy remains a major global health threat [50]. A recent systematic review highlighted that vaccine hesitancy is particularly prevalent among groups who question the necessity of vaccines, lack trust in vaccination authorities, or are unaware of the rigorous processes involved in vaccine development and their health impacts [50]. The review suggests that addressing vaccine hesitancy requires targeted education and training. This underscores the importance of incorporating vaccination information into visitor IPAC education programs, as it could help reduce the transmission of infections in LTC homes, further protecting vulnerable residents.

### 5.5. Lack of Standardized Curricula and Implementation Guidance

Another important finding of this review is the absence of standardized curricula or detailed guidance on implementing IPAC education and training for visitors. While guidelines exist, there is limited information on how these recommendations are translated into practice in individual facilities, leading to inconsistent implementation. The absence of detailed, standardized curricula may result in variations in the effectiveness of visitor education, which could undermine infection prevention efforts. This highlights the need for regulatory bodies to create clear, evidence-based, universal guidance on LTC visitor IPAC education and training to tackle inconsistencies across various areas and facilities.

### 5.6. Strengths of the Review

The timing of this review, amidst the ongoing challenges posed by COVID-19, highlights its relevance. The pandemic has raised important questions about visitor access and the role of IPAC in maintaining safe visitations during outbreaks. This review offers insights into how LTC homes can manage safe visitation while minimizing infection risks. It also provides a comprehensive overview of IPAC practices across multiple global health concerns, including influenza, SARS, Ebola, and COVID-19. By analyzing documents spanning decades, this review highlights the evolving nature of IPAC education and training for LTC visitors and identifies key gaps that need to be addressed in future research and policy.

### 5.7. Limitations

Despite the strengths of this scoping review, several limitations should be noted. One key limitation is the lack of international sources, with 84.6% of the reviewed documents originating from Canada and the U.S., leaving gaps in the information from other countries. To reduce the bias in source representation in future reviews, we will look into validated tools for translating sources reliably into English and collaborate with international researchers. The scoping review methodology also limits the ability to assess the quality of the evidence, preventing conclusions about the effectiveness of IPAC education and training. Additionally, some relevant sources may have been unintentionally excluded during screening. Lastly, while the review maps literature from 1990 to early 2023, the exclusion of more recent documents restricts its findings, as research on educating LTC visitors about IPAC has significantly increased in the past year.

### 5.8. Implications for Policy and Practice

This review highlights several important implications for policy and practice. Recently, maintaining safe visitation during infectious outbreaks, particularly during COVID-19, has become a priority. However, significant inconsistencies remain in the content, timing, frequency, and qualifications associated with delivering IPAC education and training to visitors. Given the global importance of IPAC, there is an urgent need for standardized protocols for educating LTC visitors. The lack of universally accepted guidelines presents a unique opportunity for international collaboration to share best practices, create consistency, and enhance training programs. For instance, a systematic review of LTC IPAC programs found that those incorporating educational components, along with monitoring and feedback, were effective in reducing healthcare-associated infections and promoting behavior change among healthcare workers [17]. Establishing global standards for IPAC education that includes training for visitors could ensure that all visitors are well-equipped with the knowledge necessary to minimize the spread of infections. Nursing practice is essential to achieving this, as nurses are often on the front lines, directly engaging with visitors and facilitating these important educational efforts.

### 5.9. Implications for Research

Future research should focus on the risks posed by visitors entering LTC homes during outbreaks, to inform best practices for IPAC education and training. Additionally, assessing the effectiveness of IPAC education and training in changing visitor behaviors and LTC infection rates will be important for future research. Research is needed to evaluate whether unregulated LTC staff have the skills to educate visitors, considering their infection control knowledge, access to updated resources, and ability to balance care with education responsibilities. Finally, ongoing research is essential in order to adapt IPAC training programs to evolving infectious diseases and to evaluate the sustainability of IPAC programs in LTC settings. Nursing practice is essential to achieving this, as nurses are often on the front lines, directly engaging with visitors and facilitating these important educational efforts.

## 6. Conclusions

This scoping review addresses the ongoing challenge of infection prevention in LTC homes. This review found there is no standardized approach to IPAC education and training for LTC visitors, providing an opportunity for significant policy reform and research development. The findings highlight the need to strengthen and standardize comprehensive IPAC education and training programs for LTC visitors. By addressing the gaps in current visitor education programs, this review provides a foundation for future research and policy development aimed at enhancing the safety and quality of life in LTC settings.

## Data Availability

The data presented in this study are available upon request from the corresponding authors.

## Supplementary Materials

The following supporting information can be downloaded at: https://www.mdpi.com/article/10.3390/nursrep15010017/s1, SRQR Reporting Guidelines.

## Public Involvement Statement

There was no public involvement in any aspect of this research.

## Guidelines and Standards Statement

This manuscript was drafted against the Preferred Reporting Items for Systematic reviews and Meta-Analyses extension for scoping reviews.

## Appendix A. Search Strategy

Embase (Ovid) search conducted on 15 June 2022

Date Limit: 1990 onwards

**No**	**Query**	**Results**
#1	((‘old age’ OR ‘aged care’ OR elder* OR nursing) NEAR/1 (care OR residence OR residential OR environment OR home OR facility OR setting)):ab,ti	78,055
#2	‘long term care’:ab,ti	29,246
#3	‘residential home’/exp	7951
#4	‘nursing home’/exp	60,556
#5	‘home for the aged’/exp	12,858
#6	#1 OR #2 OR #3 OR #4 OR #5	142,783
#7	handwashing:ab,ti	3859
#8	‘hand washing’:ab,ti	4255
#9	‘hand hygiene’:ab,ti	8586
#10	sanitiz*:ab,ti	3988
#11	sanitis*:ab,ti	335
#12	cleanser*:ab,ti	1694
#13	disinfect*:ab,ti	41,531
#14	glov*:ab,ti	16,240
#15	mask*:ab,ti	115,698
#16	‘patient isolat*’:ab,ti	1815
#17	‘no visit*’:ab,ti	464
#18	((guest* OR visit*) NEAR/2 (‘not allow*’ OR ‘not permit*’ OR prohibit* OR ‘closed to’)):ab,ti	115
#19	vaccin*:ab,ti	444,131
#20	((infection OR virus OR covid OR ‘covid 19’) NEAR/1 (prevent* OR mitigat* OR control* OR contain* OR manag*)):ab,ti	66,115
#21	quarantine*:ab,ti	10,420
#22	ppe:ab,ti	8022
#23	‘personal protective equipment’	9258
#24	‘glove’/exp	12,053
#25	‘mask’/exp	49,690
#26	‘patient isolation’/exp	2128
#27	‘quarantine’/exp	11,859
#28	‘infection prevention’/exp	72,778
#29	‘hand washing’/exp	18,847
#30	‘hand sanitizer’/exp	1527
#31	#7 OR #8 OR #9 OR #10 OR #11 OR #12 OR #13 OR #14 OR #15 OR #16 OR #17 OR #18 OR #19 OR #20 OR #21 OR #22 OR #23 OR #24 OR #25 OR #26 OR #27 OR #28 OR #29 OR #30	784,537
#32	volunteer*:ti,ab	281,888
#33	unpaid:ti,ab	2792
#34	‘un paid’:ti,ab	9
#35	‘non paid’:ti,ab	107
#36	‘non staff’:ti,ab	51
#37	‘non employee*’:ti,ab	57
#38	visit*:ti,ab	446,933
#39	guest*:ti,ab	21,467
#40	friend*:ti,ab	135,624
#41	famil*:ti,ab	1,531,670
#42	parent*:ti,ab	592,030
#43	mother*:ti,ab	318,044
#44	father*:ti,ab	62,242
#45	daughter*:ti,ab	34,534
#46	sibling*:ti,ab	76,960
#47	son:ti,ab	20,589
#48	sons:ti,ab	119,372
#49	brother*:ti,ab	22,333
#50	sister*:ti,ab	49,571
#51	husband*:ti,ab	23,580
#52	wife:ti,ab	8772
#53	‘significant other*’:ti,ab	5871
#54	‘spouse*’:ti,ab	25,094
#55	‘designated support*’	18
#56	‘family’/exp	578,542
#57	‘friend’/exp	23,654
#58	‘health visitor’/exp	1694
#59	‘volunteer’/exp	59,662
#60	#32 OR #33 OR #34 OR #35 OR #36 OR #37 OR #38 OR #39 OR #40 OR #41 OR #42 OR #43 OR #44 OR #45 OR #46 OR #47 OR #48 OR #49 OR #50 OR #51 OR #52 OR #53 OR #54 OR #55 OR #56 OR #57 OR #58 OR #59	3,369,651
#61	#6 AND #31 AND #60	796

Medline R (Ovid) search conducted on 15 June 2022

Date Limit: 1990 onwards

**No.**	**Query**	**Results**
1	((“old age” or “aged care” or elder* or nursing) adj1 (care or residence or residential or environment or home or facility or setting)).ab,ti.	62,785
2	“long term care”.ab,ti.	23,329
3	Long-Term Care/	27,749
4	Residential Facilities/ or Homes for the Aged/ or Nursing Homes/	48,686
5	1 or 2 or 3 or 4	122,825
6	handwashing.ab,ti.	2669
7	“hand washing”.ab,ti.	3084
8	“hand hygiene”.ab,ti.	5539
9	“sanitiz*”.ab,ti.	3416
10	“sanitis*”.ab,ti.	276
11	“cleanser*”.ab,ti.	1176
12	“disinfect*”.ab,ti.	33,926
13	“glov*”.ab,ti.	12,201
14	“mask*”.ab,ti.	92,883
15	“patient isolat*”.ab,ti.	1401
16	“no visit*”.ab,ti.	279
17	((guest* or visit*) adj2 (“not allow*” or “not permit*” or prohibit* or “closed to”)).ab,ti.	96
18	“vaccin*”.ab,ti.	371,756
19	((infection or virus or covid or “covid 19”) adj1 (prevent* or mitigat* or control* or contain* or manag*)).ab,ti.	48,381
20	“quarantine*”.ab,ti.	10,346
21	ppe.ab,ti.	6155
22	“personal protective equipment”.ab,ti.	7521
23	masks/ or gloves, protective/	8857
24	Patient Isolation/	4425
25	Quarantine/	5906
26	Infection Control/	28,378
27	Hand Disinfection/	6229
28	6 or 7 or 8 or 9 or 10 or 11 or 12 or 13 or 14 or 15 or 16 or 17 or 18 or 19 or 20 or 21 or 22 or 23 or 24 or 25 or 26 or 27	598,980
29	“volunteer*”.ab,ti.	208,286
30	unpaid.ab,ti.	2202
31	“un paid”.ab,ti.	5
32	“non paid”.ab,ti.	60
33	“non staff”.ab,ti.	34
34	“non employee*”.ab,ti.	36
35	“visit*”.ab,ti.	276,560
36	“guest*”.ab,ti.	21,026
37	“friend*”.ab,ti.	111,047
38	“famil*”.ab,ti.	1,203,235
39	“parent*”.ab,ti.	462,426
40	“mother*”.ab,ti.	245,932
41	“father*”.ab,ti.	46,440
42	“daughter*”.ab,ti.	27,679
43	“sibling*”.ab,ti.	54,316
44	son.ab,ti.	19,290
45	sons.ab,ti.	18,109
46	“brother*”.ab,ti.	14,803
47	“sister*”.ab,ti.	42,602
48	“husband*”.ab,ti.	19,794
49	“wife*”.ab,ti.	6569
50	“significant other*”.ab,ti.	4491
51	“spouse*”.ab,ti.	19,043
52	“designated support*”.ab,ti.	6
53	Friends/	6333
54	Family/	82,568
55	Visitors to Patients/	2267
56	Volunteers/	10,571
57	29 or 30 or 31 or 32 or 33 or 34 or 35 or 36 or 37 or 38 or 39 or 40 or 41 or 42 or 43 or 44 or 45 or 46 or 47 or 48 or 49 or 50 or 51 or 52 or 53 or 54 or 55 or 56	2,429,737
58	5 and 28 and 57	484

CINAHL with Full-text (EBSCOhost) search conducted on 15 June 2022

Date Limit: 1990 onwards

**No**	**Query**	**Results**
S1	TI (((“old age” or “aged care” or elder* or nursing) N1 (care or residence or residential or environment or home or facility or setting))) OR AB (((“old age” or “aged care” or elder* or nursing) N1 (care or residence or residential or environment or home or facility or setting)))	76,468
S2	TI “long term care” OR AB “long term care”	18,483
S3	(MH “Long Term Care”)	27,483
S4	(MH “Residential Care”)	7052
S5	(MH “Residential Facilities+”)	34,654
S6	S1 OR S2 OR S3 OR S4 OR S5	122,958
S7	TI handwashing OR AB handwashing	1431
S8	TI “hand washing” OR AB “hand washing”	1383
S9	TI “hand hygiene” OR AB “hand hygiene”	4125
S10	TI sanitiz* OR AB sanitiz*	849
S11	TI sanitis* OR AB sanitis*	86
S12	TI cleanser* OR AB cleanser*	537
S13	TI disinfect* OR AB disinfect*	5919
S14	TI glov* OR AB glov*	4200
S15	TI mask* OR AB mask*	19,169
S16	TI “patient isolat*” OR AB “patient isolat*”	263
S17	TI “no visit*” OR AB “no visit*”	129
S18	TI (((guest* or visit*) N2 (“not allow*” or “not permit*” or prohibit* or “closed to”))) OR AB (((guest* or visit*) N2 (“not allow*” or “not permit*” or prohibit* or “closed to”)))	354
S19	TI vaccin* OR AB vaccin*	63,340
S20	TI (((infection or virus or covid or “covid 19”) N1 (prevent* or mitigat* or control* or contain* or manag*))) OR AB (((infection or virus or covid or “covid 19”) N1 (prevent* or mitigat* or control* or contain* or manag*)))	28,224
S21	TI quarantine* OR AB quarantine*	2151
S22	TI PPE OR AB PPE	2641
S23	TI “personal protective equipment” OR AB “personal protective equipment”	3714
S24	(MH “Personal Protective Equipment”) OR (MH “Masks”) OR (MH “Gloves”)	9309
S25	(MH “Patient Isolation”)	2677
S26	(MH “Quarantine”)	1645
S27	(MH “Infection Control”) OR (MH “Handwashing”) OR (MH “Immunization”) OR (MH “Sterilization and Disinfection”)	72,053
S28	S7 OR S8 OR S9 OR S10 OR S11 OR S12 OR S13 OR S14 OR S15 OR S16 OR S17 OR S18 OR S19 OR S20 OR S21 OR S22 OR S23 OR S24 OR S25 OR S26 OR S27	168,327
S29	TI volunteer* OR AB volunteer*	48,653
S30	TI unpaid OR AB unpaid	1511
S31	TI “un paid” OR AB “un paid”	5
S32	TI “non paid” OR AB “non paid”	36
S33	TI “non staff” OR AB “non staff”	19
S34	TI “non employee” OR AB “non employee”	7
S35	TI visit* OR AB visit*	119,467
S36	TI guest* OR AB guest*	9863
S37	TI friend* OR AB friend*	41,910
S38	TI famil* OR AB famil*	309,983
S39	TI parent* OR AB parent*	161,981
S40	TI mother* OR AB mother*	94,869
S41	TI father* OR AB father*	18,984
S42	TI daughter* OR AB daughter*	6113
S43	TI sibling* OR AB sibling*	11,926
S44	TI son OR AB son	29,077
S45	TI sons OR AB sons	29,077
S46	TI brother* OR AB brother*	2767
S47	TI sister* OR AB sister*	4953
S48	TI husband* OR AB husband*	6118
S49	TI wife* OR AB wife*	2864
S50	TI “significant other*” OR AB “significant other*”	2996
S51	TI spouse* OR AB spouse*	10,129
S52	TI “designated support*” OR AB “designated support*”	3
S53	(MH “Family”)	45,323
S54	(MH “Visitors to Patients”)	2337
S55	(MH “Volunteer Workers”)	14,910
S56	S29 OR S30 OR S31 OR S32 OR S33 OR S34 OR S35 OR S36 OR S37 OR S38 OR S39 OR S40 OR S41 OR S42 OR S43 OR S44 OR S45 OR S46 OR S47 OR S48 OR S49 OR S50 OR S51 OR S52 OR S53 OR S54 OR S55	727,132
S57	S6 AND S28 AND S56	468

ERIC (EBSCOhost) search conducted on 15 June 2022

Date Limit: 1990 onwards

**No**	**Query**	**Results**
S1	TI (((“old age” or “aged care” or elder* or nursing) N1 (care or residence or residential or environment or home or facility or setting))) OR AB (((“old age” or “aged care” or elder* or nursing) N1 (care or residence or residential or environment or home or facility or setting)))	2306
S2	TI “long term care” OR AB “long term care”	828
S3	DE “Nursing Homes”	1246
S4	DE “Residential Institutions”	1087
S5	DE “Residential Care”	1268
S6	S1 OR S2 OR S3 OR S4 OR S5	5041
S7	TI handwashing OR AB handwashing	84
S8	TI “hand washing” OR AB “hand washing”	89
S9	TI “hand hygiene” OR AB “hand hygiene”	28
S10	TI sanitiz* OR AB sanitiz*	120
S11	TI sanitis* OR AB sanitis*	13
S12	TI cleanser* OR AB cleanser*	7
S13	TI disinfect* OR AB disinfect*	132
S14	TI glov* OR AB glov*	248
S15	TI mask* OR AB mask*	2295
S16	TI “patient isolat*” OR AB “patient isolat*”	1
S17	TI “no visit*” OR AB “no visit*”	6
S18	TI (((guest* or visit*) N2 (“not allow*” or “not permit*” or prohibit* or “closed to”))) OR AB (((guest* or visit*) N2 (“not allow*” or “not permit*” or prohibit* or “closed to”)))	94
S19	TI vaccin* OR AB vaccin*	707
S20	TI (((infection or virus or covid or “covid 19”) N1 (prevent* or mitigat* or control* or contain* or manag*))) OR AB (((infection or virus or covid or “covid 19”) N1 (prevent* or mitigat* or control* or contain* or manag*)))	397
S21	TI quarantine* OR AB quarantine*	100
S22	TI PPE OR AB PPE	61
S23	TI “personal protective equipment” OR AB “personal protective equipment”	65
S24	S7 OR S8 OR S9 OR S10 OR S11 OR S12 OR S13 OR S14 OR S15 OR S16 OR S17 OR S18 OR S19 OR S20 OR S21 OR S22 OR S23	4262
S25	TI volunteer* OR AB volunteer*	13,750
S26	TI unpaid OR AB unpaid	552
S27	TI “un paid” OR AB “un paid”	3
S28	TI “non paid” OR AB “non paid”	14
S29	TI “non staff” OR AB “non staff”	6
S30	TI “non employee” OR AB “non employee”	5
S31	TI visit* OR AB visit*	21,111
S32	TI guest* OR AB guest*	2129
S33	TI friend* OR AB friend*	19,941
S34	TI famil* OR AB famil*	128,316
S35	TI parent* OR AB parent*	126,111
S36	TI mother* OR AB mother*	25,378
S37	TI father* OR AB father*	9012
S38	TI daughter* OR AB daughter*	2539
S39	TI sibling* OR AB sibling*	4082
S40	TI son OR AB son	2449
S41	TI sons OR AB sons	2449
S42	TI brother* OR AB brother*	1412
S43	TI sister* OR AB sister*	1355
S44	TI husband* OR AB husband*	1933
S45	TI wife* OR AB wife*	1261
S46	TI “significant other*” OR AB “significant other*”	957
S47	TI spouse* OR AB spouse*	2480
S48	TI “designated support*” OR AB “designated support*”	4
S49	DE “Family (Sociological Unit)” OR DE “African American Family” OR DE “Daughters” OR DE “Dependents” OR DE “Heads of Households” OR DE “Homemakers” OR DE “One Parent Family” OR DE “Parents” OR DE “Siblings” OR DE “Sons” OR DE “Spouses” OR DE “Widowed”	26,255
S50	DE “Volunteers” OR DE “Student Volunteers”	6442
S51	S25 OR S26 OR S27 OR S28 OR S29 OR S30 OR S31 OR S32 OR S33 OR S34 OR S35 OR S36 OR S37 OR S38 OR S39 OR S40 OR S41 OR S42 OR S43 OR S44 OR S45 OR S46 OR S47 OR S48 OR S49 OR S50	277,745
S52	S6 AND S24 AND S51	23

AgeLine (EBSCOhost) search conducted on 14 December 2022

Date Limit: 1990 onwards

**No**	**Query**	**Limiters/** **Expanders**	**Last Run Via**	**Results**
S1	(((TI (((“old age” or “aged care” or elder* or nursing) N1 (care or residence or residential or environment or home or facility or setting))) OR AB (((“old age” or “aged care” or elder* or nursing) N1 (care or residence or residential or environment or home or facility or setting)))) OR (TI “long term care” OR AB “long term care”))) AND ((TI handwashing OR AB handwashing) OR (TI “hand washing” OR AB “hand washing”) OR (TI “hand hygiene” OR AB “hand hygiene”) OR (TI sanitiz* OR AB sanitiz*) OR (TI sanitis* OR AB sanitis*) OR (TI cleanser* OR AB cleanser*) OR (TI disinfect* OR AB disinfect*) OR (TI glov* OR AB glov*) OR (TI mask* OR AB mask*) OR (TI “patient isolat*” OR AB “patient isolat*”) OR (TI “no visit*” OR AB “no visit*”) OR (TI (((guest* or visit*) N2 (“not allow*” or “not permit*” or prohibit* or “closed to”))) OR AB (((guest* or visit*) N2 (“not allow*” or “not permit*” or prohibit* or “closed to”)))) OR (TI vaccin* OR AB vaccin*) OR (TI (((infection or virus or covid or “covid 19”) N1 (prevent* or mitigat* or control* or contain* or manag*))) OR AB ( ((infection or virus or covid or “covid 19”) N1 (prevent* or mitigat* or control* or contain* or manag*)))) OR (TI quarantine* OR AB quarantine*) OR (TI PPE OR AB PPE) OR (TI “personal protective equipment” OR AB “personal protective equipment”))) AND ((TI volunteer* OR AB volunteer*) OR (TI unpaid OR AB unpaid) OR (TI “un paid” OR AB “un paid”) OR (TI “non paid” OR AB “non paid”) OR (TI “non staff” OR AB “non staff”) OR (TI “non employee” OR AB “non employee”) OR (TI visit* OR AB visit*) OR (TI guest* OR AB guest*) OR (TI friend* OR AB friend*) OR (TI famil* OR AB famil*) OR (TI parent* OR AB parent*) OR (TI mother* OR AB mother*) OR (TI father* OR AB father*) OR (TI daughter* OR AB daughter*) OR (TI sibling* OR AB sibling*) OR (TI son OR AB son) OR (TI brother* OR AB brother*) OR (TI sister* OR AB sister*) OR (TI husband* OR AB husband*) OR (TI wife* OR AB wife*) OR (TI “significant other*” OR AB “significant other*”) OR (TI spouse* OR AB spouse*) OR (TI “designated support*” OR AB “designated support*”)))	Expanders—Apply equivalent subjects Search modes—Boolean/Phrase	Interface—EBSCOhost Research Databases Search Screen—Advanced Search	1757

## Appendix B. Grey Literature Search Strategy

**Table A1:** Grey Literature Search of Long-Term Care Home Associations.

Association	Country	Relevant Documents Identified
Aged and Community Services Australia	Australia	
Aged Care Guild	Australia	
Agency for Integrated Care	Singapore	Agency for Integrated Care Caregiver Training Course Summary
Alberta Caregivers Association	Canada	
Alberta Continuing Care Association	Canada	
American Health Care Association	USA	Sample Policy for Emergent Infectious Diseases for Skilled Nursing Care Centers
Assisted Living Federation of America	USA	
Association of Healthcare Providers India	India	
British Columbia Care Providers Association	Canada	
Canadian Association for Long Term Care	Canada	
Canadian Caregiver Coalition	Canada	
Care England	England	
Caregiver Action Network	USA	
Caregiver India Foundation	India	
Caregiver Saathi	India	
Caregivers Alberta	Canada	
Caregivers Association of Nigeria	Nigeria	
Caregivers Nova Scotia	Canada	
Carers Alliance	Hong Kong	
Carers Australia	Australia	
Carers NSW	Australia	
Carers NZ	New Zealand	
Carers Queensland	Australia	
Carers Trust	United Kingdom	
Carers UK	United Kingdom	
Carers Victoria	Australia	
Centers for Medicare and Medicaid Services	USA	
Eldercare Locator	USA	
Family Caregiver Alliance	USA	
Family Caregivers of British Columbia	Canada	
Family Carers Ireland	Ireland	
HelpAge India	India	
Irish Association of Social Care Worker	Ireland	
Leading Age Services Australia	Australia	
LeadingAge	USA	
Long-Term Care Ombudsman Program	USA	
Malaysian Caregivers Association	Malaysia	
Manitoba Association of Residential and Community Care	Canada	
National Alliance for Caregiving	USA	
National Association of Aged Care Providers	Australia	
National Care Association	England	
National Center for Assisted Living	USA	
National Consumer Voice for Quality Long-Term Care	USA	
New Brunswick Association of Nursing Homes	Canada	
New Zealand Aged Care Association	New Zealand	
Northern Ireland Association of Homes for the Aged	Ireland	
Nursing Homes Ireland	Ireland	
Nursing Homes of Nova Scotia Association	Canada	
Ontario Caregiver Organization	Canada	
Ontario Long Term Care Association	Canada	
PEI Association for Community Long Term Care Homes	Canada	
PEI Association of Licensed Community Care Facilities	Canada	
Saskatchewan Association of Long-Term Care Providers	Canada	
Scottish Care	Scotland	
Seniors Newfoundland and Labrador	Canada	
South African Care Forum	South Africa	
South African Care Forum	South Africa	
South African Care Workers Association	South Africa	
The Princess Royal Trust for Carers	United Kingdom	
Well Spouse Association	USA	
Yukon Department of Health and Social Services	Canada	
Total Documents Identified		2

**Table A2:** Grey Literature Search of Long-Term Care Home Organizations and Research Centers.

Organization	Country	Relevant Documents Identified
Agency for Research in Healthcare Quality	USA	
Australian Association of Gerontology	Australia	
Australian Institute of Health and Welfare	Australia	
Brown University Center for Gerontology and Healthcare Research	USA	
Canadian Centre for Elder Law	Canada	
Canadian Institute for Health Information	Canada	
Centers for Disease Control and Prevention	USA	IPAC Recommendations for LTC Updated 2023
Centre for Excellence in Population Ageing Research	Australia	
Centre for Gerontology and Rehabilitation	Ireland	
Centre for Learning, Research and Innovation in LTC	Canada	New IPAC eLearning course
Institute for Health System Solutions and Virtual Care	Canada	
Institute for Research on Aging	Canada	
International Long Term Care Policy Network	International	
Irish Centre for Social Gerontology	Ireland	
Irish Longitudinal Study on Ageing	Ireland	
Manitoba Centre for Health Policy	Canada	
Marcus Institute for Aging Research at Hebrew SeniorLife	USA	
Massey University, Health and Ageing Research Team	New Zealand	
National Ageing Research Institute	Australia	
National Institute on Aging	Canada	
National Institute on Aging	USA	
New Zealand Aged Care Association Research Center	New Zealand	
Rand Corporation Center for the Study of Aging	USA	
South African Medical Research Council	South Africa	
Stanford Center on Longevity	USA	
University of Auckland, School of Nursing	New Zealand	
University of Cape Town, Division of Geriatric Medicine	South Africa	
University of East Anglia, Centre for Research on Ageing and Gender	England	
University of Leeds, Centre for Research in Nursing and Midwifery	England	
University of Otago, New Zealand Institute for Research on Aging	New Zealand	
University of Oxford, Oxford Institute of Population Ageing	England	
University of Sheffield, School of Nursing and Midwifery	United Kingdom	
University of Southampton, Centre for Research on Ageing	England	
World Health Organization	International	Infection Prevention and Control for Long-Term Care Facilities in the Context of Covid 19
Total Documents Identified		3

Note. USA = United States of America; LTC = Long-term care; IPAC = Infection prevention and control.

**Table A3:** Grey Literature Obtained via the Google Search Engine.

Website	Country	Relevant Documents Identified
Australian Government	Australia	Visiting an aged care home during an outbreak
Centers for Disease Control and Prevention	Canada	Core Infection Prevention and Control Practices for Safe Healthcare Delivery in All Settings
Fraser Health	Canada	Infection Control Manual—Residential Care
Ministry of Long-Term Care	Canada	Infection prevention and control program guidance
Missouri Department of Health Services	USA	Infection Control Guidelines for Long-Term Care Facilities
Ontario Health	Canada	Infection Prevention and Control Standard for Long-Term Care Homes
Ontario Public Health	Canada	Visitors’ policy in long-term care homes during COVID-19 pandemic
Provincial Infection Control Network	Canada	Residential care infection prevention and control manual.
Public Health Agency of Canada	Canada	Prevention and Control of Influenza during a Pandemic for All Healthcare Settings
Public Health Agency of Canada	Canada	The Chief Public Health Officer’s Report on the State of Public Health in Canada 2013
Public Health Agency of Canada	Canada	Clostridium Difficile Infection
Public Health Agency of Canada	Canada	Routine Practices and Additional Precautions for Preventing the Transmission of Infection in Healthcare Settings Part B
Public Health Agency of Canada	Canada	Infection prevention and control for COVID-19: Interim guidance for long-term care homes
ResCare Community Living	USA	Patient & Family Education Package
UniversalCare	Canada	Infection Prevention and Control Policy
Total Documents Identified		15

Note. USA = United States of America.

## Appendix C. Characteristics of Included Studies

**Table A4:** Document Characteristics for IPAC Education and Training for LTC Visitors (N = 26).

Author, Year	Country	Type	Audience/Sample	Setting(s)	Phenomena/Purpose	Relevant Findings/Recommendations
Agency for IntegratedCare, 2023 [32]	SG	Education Resource	Family	LTCF	Training course on providing safe care.	The course requires attendees to understand standard precautions to prevent infection spread.
American Healthcare Association, 2015 [35]	US	Policy	Healthcare workers	LTCF	Guide preparation for infectious diseases.	Families should be provided with education about the outbreak and the center’s response strategy.
Australian Government, 2021 [22]	AU	Guideline	Healthcare workers	LTCF	Guide IPAC practices based on COVID-19 status in the community.	Visitors are essential to aged care to prevent resident deconditioning. IPAC education should be provided to visitors during all phases of COVID-19 outbreaks.
Australian Government, 2023 [34]	AU	Education Resources	Visitors	LTCF	Education on key practices to carry out when visiting LTC during an infectious outbreak.	Visitors are prompted on what to do before, during, and after a visit for safety during an infectious outbreak.
Augustin & Barry, 2021 [39]	CA	Position Statement	IPAC Professionals	LTCF	Describe and recommended IPAC practices.	IPAC education should be provided to all visitors of LTC homes.
Bergman, 2020 [42]	US; CA	Consensus Paper	Experts—LTC	LTCF	Generate consensus to guide visitation by essential family caregivers and visitors during the COVID-19 pandemic.	Consensus was reached on 12 statements related to visitor guidance including IPAC strategies.
CDC, 2022 [28]	US	Guideline	Healthcare Organizations/ Policy Makers	All *	Core IPAC guidelines for safe healthcare delivery in all healthcare settings.	Visitors to all healthcare facilities should be provided with IPAC education.
CDC, 2023 [29]	US	Guideline	Healthcare workers	All	Introduce a framework to guide selection and implementation of specific IPAC practices based on individual circumstances (e.g., universal source control).	Facilities should provide instruction before a visitor enters a patient’s room on IPAC practices and should refrain from visiting if sick.
Centers for Learning, Research, &Innovation, 2023 [33]	CA	Education Resource	Healthcare workers/ Visitors	LTCF	Educate and practice applying IPAC principles to care.	Focus on breaking the chain of transmission through routine practices, best practices, and hand hygiene.
Fraser Health, 2013 [25]	CA	Guideline	Healthcare workers	LTCF	Guidelines for IPAC for residential care.	Visitors should be educated on IPAC
Missouri Health & Senior Services, 2005 [23]	US	Guideline	Healthcare workers	LTCF	Guide for establishing high-quality IPAC in MDHS LTC.	Visitor education is recommended when there is a suspected or known disease or organism in the facility.
Ontario Health, 2022 [40]	CA	Standard	IPAC Professionals/ LTCF	LTCF	Standard of current evidence-based IPAC practices in LTC.	LTC should have IPAC professional and IPAC program that includes educating visitors.
Ontario Ministry of Long-Term Care, 2021 [26]	CA	Guideline	LTCF	LTCF	Guide implementation of IPAC programs in LTC.	IPAC programs need to contain education for visitors.
Ontario Public Health, 2022 [38]	CA	Policy	Healthcare workers /Visitors	LTCF	Visitor policy during COVID-19 pandemic.	All visitors to Ontario LTC homes are to be educated on IPAC practices.
PHAC, 2011 [4]	CA	Guideline	Healthcare workers	All	Guide IPAC and occupational health planning and management of pandemic Influenza.	During an influenza outbreak, visitors should only visit LTC if they’ve already had influenza or were immunized. If visitors have symptoms, they should be educated.
PHAC, 2013 [38]	CA	Report	Healthcare workers	All	Describe state of HAIs; educate, raise awareness, and provide recommendations to prevent HAIs.	80% of infections are spread by visitors, patients, and healthcare workers (ie., MRSA, *C. difficile, staph*). Facilities need to educate visitors on IPAC.
PHAC, 2013 [3]	CA	Guideline	Healthcare workers	LTCF	Guide IPAC for management of residents with *C. difficile* infection.	Recommend visitors be educated about the IPAC precautions in place. Any visitor participating in resident care should be educated on personal-protective equipment (PPE).
PHAC, 2017 [5]	CA	Guideline	Healthcare workers/ IPAC Professionals	All	Guide routine practices and additional precautions for preventing transmission of HAIs.	Healthcare workers should educate visitors on IPAC practices as indicated. Visitors participating in care should be educated about PPE.
PHAC, 2021 [27]	CA	Guideline	LTCF	LTCF	Update and guide IPAC for COVID-19.	Visitors should be instructed on IPAC practices and refrain from visiting if sick.
Provincial Infection Control Network, 2011 [24]	CA	Guideline	LTCF	LTCF	Guide LTC homes on the current best practices for preventing and controlling infections.	The basis of good IPAC practice is through educating staff, residents, and visitors.
ResCare CommunityLiving, 2020 [31]	US	Education Resources	Visitors	Alternate Level of Care Facilities	Describe and evaluate methods to mitigate the spread of COVID-19, e.g., visitor education resources.	Visitor education package provides information on COVID-19 IPAC practices.
Siegel, 2023 [8]	US	Guideline	Healthcare workers/ Policy Makers	All	Guide isolation precautions, IPAC program development, implementation, and evaluation.	Visitors are sources of many HAIs (i.e., pertussis, influenza, and SARS-CoV). Visitors should be educated on IPAC practices.
Stefanacci, 2020 [41]	US	Opinion Paper	Policy Makers	LTCF	Discuss a 4S process for safer visitations based on CDC recommendations.	The 4S process for safer visitations to LTC includes scheduling and education, screening, social distancing. and PPE, and an outside setting.
Tupper, 2020 [1]	CA	Opinion Paper	Policy Makers	LTCF	Describe importance of visitors and indications for clinical practice.	Prioritization of IPAC without ensuring resident psychosocial needs are protected is a short-sighted approach that will lead to harm.
UniversalCare, 2022 [36]	CA	Policy	LTCF	LTCF	Policy for pandemics, epidemics, and outbreaks.	Visitors to LTC homes need to be educated and trained on IPAC and be compliant with practices.
WHO, 2021 [30]	INT	Guideline	Healthcare workers/ Visitors	LTCF	Guide IPAC to prevent COVID-19 and support safe visiting for residents’ well-being.	Adequate visitor IPAC training and education by an IPAC professional is essential to reduce the risk of COVID-19 among LTC residents.

Note. CDC = Centers for Disease Control and Prevention; WHO = World Health Organization; SG = Singapore; CA = Canada, US = United States of America; AU = Australia; INT = International; LTCF = Long-term care facilities; IPAC = Infection prevention and control. * “All” settings includes long-term care and acute care.
