# Supplementary material for: Education and Training on Infection Prevention and Control Provided by Long-Term Care Homes to Visitors: A Scoping Review
Source: Nurs Rep. 2025 Jan 10;15(1):17. doi: 10.3390/nursrep15010017 (PMC11767841; doi:10.3390/nursrep15010017)
Supplement: Supplementary file 1 [file nursrep-15-00017-s001.zip › nursrep-3399957-supplementary.docx]

**Supplementary materials**

**Detailed description of the findings**

For more information on the study, please contact Rachel MacLean at rachel.maclean@unb.ca . A detailed description of the findings can be found in the following dissertation:

MacLean, RFH. *Education and training for infection prevention and control provided by*

*long-term care homes to visitors: A scoping review in partnership with Loch Lomond*

*Villa.* Dissertation. University of New Brunswick; 2024.

**Table S1**

*Data extraction table*

| Author, Year |  |
| --- | --- |
| Country |  |
| Study aim |  |
| Type of study/document |  |
| LTC description |  |
| Education/training content included |  |
| Mode of delivery |  |
| Frequency of delivery |  |
| Timing of delivery |  |
| Where education/training occurred |  |
| Individuals and qualifications responsible for the education/training |  |
| How has the education/training evolved over time? |  |

**Studies ineligible following full-text review**

1. Ahc M. Wash your hands—or people die. *Hosp Infect Control Prev*. 2018;45(4):12. <https://www.reliasmedia.com/articles/142418-wash-your-hands-or-people-die>.

**Exclusion reason: Ineligible phenomenon**

1. Al Hamad H, Malkawi MMM, Nooh M, Al-Mutawa JH, Doiphode SH, Sathian B. Investigation of a COVID-19 outbreak and its successful containment in a long-term care facility in Qatar. *Eur Geriatr Med.* 2021;12(Suppl 1):S177.

**Exclusion reason: Ineligible phenomenon**

1. Al Hamad H, Malkawi MMM, Al Ajmi JAAA, Al-Mutawa MNJH, Doiphode SH, Sathian B. Investigation of a COVID-19 outbreak and its successful containment in a long-term care facility in Qatar. *Front Public Health*. 2021;9:1-11.
   **Exclusion reason: Ineligible phenomenon**
2. American Healthcare Association. Infection prevention and control for long-term care facilities: handbook. Demare Thompson Learning: CA, USA; 1997.
   **Exclusion reason: Unable to obtain full-text**
3. Australian Nursing & Midwifery Federation. The impact of COVID-19 on aged care. *Aust Nurs Midwifery J*. 2020;26(11):16.
   **Exclusion reason: Ineligible phenomenon**
4. Aykaç N, Yüksel Eryiğit Ö, Elbek O. Evaluation of the measures taken in nursing homes of the Istanbul metropolitan municipality during the COVID-19 pandemic. *Turk Geriatri Dergisi*. 2021;24(1):13-22.
   **Exclusion reason: Ineligible phenomenon**
5. Backhaus R, Verbeek H, de Boer B, Ulrings JHJ, Gerritsen DL, Koopmans RTCM, Hamers JPH. From wave to wave: a Dutch national study on the long-term impact of COVID-19 on well-being and family visitation in nursing homes*. BMC Geriatr*. 2021;21(1):1-7.
   **Exclusion reason: Ineligible phenomenon**
6. Bolcato M, Aurilio M, Di Mizio G, Piccioni A, Feola A, Bonsignore A, et al. The difficult balance between ensuring the right of nursing home residents to communication and their safety*. Int J Environ Res Public Health.* 2021;18(5):2484.
   **Exclusion reason: Ineligible phenomenon**
7. Bouchoucha SL, Bloomer MJ. Family‐centered care during a pandemic: The hidden impact of restricting family visits. *Nurs Health Sci*. 2021;23(1):4-6.
   **Exclusion reason: Ineligible population**
8. Chock L. Norovirus outbreak in a long-term care facility*. Am J Infect Control*. 2012;40(5):e110-e111.
   **Exclusion reason: Conference proceeding**
9. Christ G. CMS loosens visitation restrictions for nursing homes. *Mod Healthc.* 2021;51(11):7.
   **Exclusion reason: Ineligible phenomenon**
10. Chung CH, Li YC, Wang YF, Liu YC, Lin WC. Influenza A outbreak investigation and control measures of regional teaching hospital*. Int J Antimicrob Agents*. 2017;50:S144.
    **Exclusion reason: Ineligible phenomenon**
11. Evans G. Clock starts ticking when COVID-19 enters nursing home: Look for any early signs and symptoms. *Hosp Infect Control Prev*. 2020;47(6):1-2.
    **Exclusion reason: Ineligible phenomenon**
12. Fletcher KR, Cinalli M. Identification, optimal management, and infection control measures for Clostridium difficile-associated disease in long-term care. *Geriatr Nurs*. 2007;28(3):171-181.
    **Exclusion reason: Ineligible phenomenon**
13. Frazer K, Mitchell L, Stokes D, Lacey E, Crowley E, Kelleher C. A rapid systematic review of measures to protect older people in long-term care facilities from COVID-19. *BMJ Open*. 2021;11(10):e047012.
    E**xclusion reason: Ineligible phenomenon**
14. Gilissen J, Pivodic L, Unroe KT, Van den Block L. International COVID-19 palliative care guidance for nursing homes leaves key themes unaddressed*. Palliat Care Soc Pract*. 2021;60(2):e56-69.
    **Exclusion reason: Ineligible phenomenon**
15. Gussin G, Singh R, Gohil SK, Saavedra R, Tjoa T, Pedroza R, Berman C, Park J, Ghasemian K, Osalvo A, Hsi J, Hsi EA, Chun S, Zahn M, Fonda E, Huang SS. Impact of nursing home universal decolonization and COVID prevention training on COVID-19 burden during the 2020-2021 winter surge in Orange County, California. *Open Forum Infect Dis.* 2021;8(Suppl 1):S32.
    **Exclusion reason: Ineligible phenomenon**
16. Hado E, Friss Feinberg L. Amid the COVID-19 pandemic, meaningful communication between family caregivers and residents of long-term care facilities is imperative. *J Aging Soc Policy.* 2020;32(4/5):410-5.
    **Exclusion reason: Ineligible phenomenon**
17. Ham C, Montgomery P. Exploring infection prevention challenges and opportunities in adult family homes. *Am J Infect Control*. 2021;49(6):S6-S7.
    **Exclusion reason: Ineligible phenomenon**
18. Hartigan I, Kelleher A, McCart J, Cornally N. Visitor restrictions during the COVID-19 pandemic: An ethical case study. *Nurs Ethics*. 2021;28(7-8):1111-1123.
    **Exclusion reason: Ineligible phenomenon**
19. Haugen DF, Romarheim E, Solvåg K, Sigurdardottir KR. Care for dying patients under the COVID-19 pandemic in Norway: A survey of bereaved relatives. *Palliat Med*. 2021;35(1 Suppl):34.
    **Exclusion reason: Conference proceedings**
20. Massachusetts HCPro. COVID-19: What SNFs need to know. Billing alert for long-term care. 2020;22(5):4-6.
    **Exclusion reason: Ineligible phenomenon**
21. Ingram C, Downey V, Roe M, Chen Y, Archibald M, Kallas K-A, Kumar J, Naughton P, Uteh CO, Rojas-Chaves A, Shrestha S, Syed S, Büttner FC, Buggy C, Perrotta C. COVID-19 prevention and control measures in workplace settings: A rapid review and meta-analysis. *Int J Environ Res Public Health.* 2021;18:7847.
    **Exclusion reason: Ineligible phenomenon**
22. Ingram C, Downey V, Roe M, Chen Y, Cléirigh Büttner F, Buggy C, et al. COVID-19 prevention and control measures in workplace settings: A rapid review and meta-analysis. *Eur J Public Health*. 2021;31:iii113-iii.
    **Exclusion reason: Ineligible phenomenon**
23. Iwamoto Sr P, Selvage D. Control and containment of a norovirus outbreak in a skilled nursing facility unit. *Am J Infect Control*. 2013;41(6):S135.
    **Exclusion reason: Ineligible phenomenon**
24. Jin C. Working together in Seattle, Washington: Impact of a collaboration of Providence hospice team and long-term care facility with COVID-19 outbreak on patient care. *J Pain Symptom Manag.* 2021;61(3):659.
    **Exclusion reason: Ineligible phenomenon**
25. Kazawa K, Kodama A, Sugawara K, Hayashi M, Ota H, Son D, et al. Person-centered dementia care during COVID-19: A qualitative case study of impact on and collaborations between caregivers. *BMC Geriatr.* 2022;22(1):1-11.
    **Exclusion reason: Ineligible phenomenon**
26. Kim JJ, Coffey KC, Morgan DJ, Roghmann JC. Nursing home visitation restrictions during COVID-19—Balancing compassion and safety. *Am J Infect Control*. 2021;49(3):407.
    **Exclusion reason: Ineligible phenomenon**
27. Koopmans R, Verbeek H, Bielderman A, Janssen M, Persoon A, Lesman-Leegte I, et al. Reopening the doors of Dutch nursing homes during the COVID-19 crisis: Results of an in-depth monitoring. *Int Psychogeriatr*. 2022;24(4):391-398.
    **Exclusion reason: Ineligible phenomenon**
28. Leaver M. Guest editorial. Progress towards infection control programs in residential aged care facilities*. Aust Infect Control*. 2001;6(4):108-109.
    **Exclusion reason: Ineligible phenomenon**
29. Lee DTF, Yu DSF, Ip M, Tang JYM. Implementation of respiratory protection measures: Visitors of residential care homes for the elderly. *Am J Infect Control*. 2017;45(2):197-199.
    **Exclusion reason: Ineligible phenomenon**
30. Loizeau A, D’Agata E, Shaffer M, et al. The trial to reduce antimicrobial use in nursing home residents with Alzheimer's disease and other dementias: Study protocol for a cluster randomized controlled trial. *Trials.* 2019;20(1).

**Exclusion reason: Not education & training of family.**

1. Low LF, Hinsliff-Smith K, Sinha SK, et al. Safe visiting is essential for nursing home residents during the COVID-19 pandemic: An international perspective*. J Am Med Dir Assoc.* 2021;22(5):977-978.
   **Exclusion reason: Not education & training of family.**
2. MacDonald CJ, Walton R. E-learning education solutions for caregivers in long term care (LTC) facilities: New possibilities*. Educ Health (Abingdon).* 2007;20(3):2007.
   **Exclusion reason: Does not address IPC measures.**
3. McMichael TM, Clark S, Pogosjans S, et al. COVID-19 in a long-term care facility - King County, Washington, February 27-March 9, 2020*. MMWR Morb Mortal Wkly Rep*. 2020;69(12):339-342.
   **Exclusion reason: Not education & training of family.**
4. Meehan A, Uth R, Gadbois E, et al. Impact of COVID-19 on infection control practices in skilled nursing facilities. *JAMA.* 2022;23(3):18.
   **Exclusion reason: Not education & training of family.**
5. Mendelsohn L, McCleary L. Reflections on working with families in long term care during the SARS restrictions. *Perspect* (Gerontol Nurs Assoc (Canada)). 2003;27(3):8-12. **Exclusion reason: Not education & training of family.**
6. Mitchell LL, Albers EA, Birkeland RW, et al. Caring for a relative with dementia in long-term care during COVID-19. *J Am Med Dir Assoc.* 2022;23(3):428.
   **Exclusion reason: Not education & training of family.**
7. Mollon N. Infection control assessment and response (ICAR) in Michigan: Past, present and future. *Am J Infect Control.* 2019;47(6):35.
   **Exclusion reason: Not education & training of family.**
8. Morgan J, Lavin MA. The infection control assessment and response tool: Is it useful during an outbreak? *Am J Infect Control.* 2016;44(6):125.
   **Exclusion reason: Not education & training of family.**
9. Moussa CA. Lights, camera, action! Development of a new educational video for long term care. *Am J Infect Control.* 2009;37(5):154.
   **Exclusion reason: Not education & training of family.**
10. Nash WA, Harris LM, Heller KE, Mitchell BD. "We are saving their bodies and destroying their souls.": Family caregivers' experiences of formal care setting visitation restrictions during the COVID-19 pandemic. *J Age Soc Policy.* 2021;33(4-5):398-413.
    **Exclusion reason: Does not address IPC measures.**
11. Nemann A, Martin B. Pandemic support: The coronavirus may have turned our world upside down, but there are things you can do to keep yourself and your family healthy and safe. *PN.* 2020;74(5):22-26.
    **Exclusion reason: Wrong patient population.**
12. Nguyen LKN, Howick S, McLafferty D, et al. Impact of visitation and cohorting policies to shield residents from COVID-19 spread in care homes: an agent-based model*. Am J Infect Control.* 2021;49(9):1105-1112.
    **Exclusion reason: Not education & training of family.**
13. Ostrowsky B, Weil L, Olaisen R, et al. Real-time virtual infection prevention and control assessments in skilled nursing homes, New York, March 2020 - A pilot project. *Infect Control Hosp Epidemiol*. 2022;43(3):351-357.
    **Exclusion reason: Not education & training of family.**
14. Petrovic M, Hickman S, Mack L, Unroe K. Re-opening nursing facilities to visitors during a pandemic: An early look at experiences. *J Am Geriatr Soc.* 2021;69(1):303.
    **Exclusion reason: Not education & training of family.**
15. Rios P, Radhakrishnan A, Williams C, et al. Preventing the transmission of COVID-19 and other coronaviruses in older adults aged 60 years and above living in long-term care: A rapid review. *Syst Rev.* 2020;9(1).
    **Exclusion reason: Not education & training of family.**
16. Rummukainen M, Jakobsson A, Karppi P, Kautiainen H, Lyytikäinen O. Promoting hand hygiene and prudent use of antimicrobials in long-term care facilities*. Am J Infect Control.* 2009;37(2):168-171.
    **Exclusion reason: Not education & training of family.**
17. Schlaudecker JD. Essential family caregivers in long-term care during the COVID-19 pandemic. *JAMA.* 2020;21(7):983.
    **Exclusion reason: Does not address IPC measures.**
18. Seiffert P, Kłosińska U, Słowiński R, Maślanka-Seiffert B, Derejczyk G, Derejczyk J. The implementation of Polish national recommendations against COVID-19 outbreak in long-term care facilities: A case study. *Eur Geriatr Med*. 2020;11(1):49.
    **Exclusion reason: Not education & training of family.**
19. Shrader CD, Assadzandi S, Pilkerton CS, Ashcraft AM. Responding to a COVID-19 outbreak at a long-term care facility. *J Appl Gerontol.* 2021;40(1):14-17.
    **Exclusion reason: Not education & training of family.**
20. Simard J, Volicer L. Loneliness and isolation in long-term care and the COVID-19 pandemic. *J Am Med Dir Assoc.* 2020;21(7):966-967.
    **Exclusion reason: Does not address IPC measures.**
21. Sizoo EM, Monnier AA, Bloemen M, Hertogh CMPM, Smalbrugge M. Dilemmas with restrictive visiting policies in Dutch nursing homes during the COVID-19 pandemic: A qualitative analysis of an open-ended questionnaire with elderly care physicians*. J Am Med Dir Assoc.* 2020;21(12):1774.
    **Exclusion reason: Not education & training of family.**
22. Smaling HJA, Tilburgs B, Achterberg WP, Visser M. The impact of social distancing due to the COVID-19 pandemic on people with dementia, family carers, and healthcare professionals: A qualitative study. *Int J Environ Res Public Health.* 2022;19(1).
    **Exclusion reason: Does not address IPC measures.**
23. Stone P, Herzig C, Pogorzelska-Maziarz M, et al. Understanding infection prevention and control in nursing homes: A qualitative study. *Geriatr Nurs.* 2015;36(4):267-272. **Exclusion reason: Not education & training of family.**
24. Tan LF, Tan MF. Pandemic to endemic: New strategies needed to limit the impact of COVID-19 in long-term care facilities (LTCFs). *J Am Geriatr Soc.* 2022;70(1):72-73. **Exclusion reason: Not education & training of family.**
25. Tretteteig S, Eriksen S, Hillestad AH, et al. The experience of relatives of nursing home residents with COVID-19: A qualitative study. *Nurs Res Rev.* 2022;12:17-27.
    **Exclusion reason: Not education & training of family.**
26. Usher K, Durkin J, Gyamfi N, Warsini S, Jackson D. Preparedness for viral respiratory infection pandemic in residential aged care facilities: A review of the literature to inform post-COVID-19 response. *J Clin Nurs.*
    **Exclusion reason: Not education & training of family.**
27. Van Tol L, Smaling H, Groothuijse J, et al. COVID-19 management in nursing homes by outbreak teams (MINUTES) - Study description and data characteristics: A qualitative study. *BMJ Open.* 2021;11(11):e053235.
    **Exclusion reason: Not education & training of family.**
28. Verbeek H, Gerritsen D, Backhaus R, et al. Allowing visitors back in the nursing home during the COVID-19 crisis: A Dutch national study into first experiences and impact on well-being*. J Am Med Dir Assoc.* 2020;21(7):900-904.
    **Exclusion reason: Not education & training of family.**
29. Wagner JM, Negley J, Towery S, Stanfill C, Lam K. Effective public health response in carbapenemase-producing carbapenem-resistant enterobacteriaceae (CP-CRE) outbreak in a Georgia long-term care facility. *Am J Infect Control.* 2019;47(6):S33.
    **Exclusion reason: Not education & training of family.**
30. Wang LL, DeWitt D, Ping LC, et al. Mitigation of COVID-19 risk among older adults in nursing homes: A public survey. *J Gerontol Nurs.* 2021;47(3):23-28.
    **Exclusion reason: Not education & training of family.**
31. Wang L, Qi N, Zhou Y, Zhang H. Prevention and infection control of COVID-19 in nursing homes: Experience from China. *Age Ageing.* 2020;49(6):894-895.
    **Exclusion reason: Not education & training of family.**
32. Wilfinger C. Infection prevention cohort unit: A strategy to promote patient safety. *Am J Infect Control.* 2009;37(5):E110-E111.
    **Exclusion reason: Unable to obtain full-text.**
33. World Health Organization. Infection prevention and control guidance for long-term care facilities in the context of COVID-19: Interim guidance. WHO; March 2020. **Exclusion reason: Unable to obtain full-text.**

**Table S2**

Preferred Reporting Items for Systematic reviews and Meta-Analyses extension for Scoping Reviews (PRISMA-ScR) Checklist

| **SECTION** | **ITEM** | **PRISMA-ScR CHECKLIST ITEM** | **REPORTED ON PAGE #** |
| --- | --- | --- | --- |
| **TITLE** | | | |
| Title | 1 | Identify the report as a scoping review. | 1 |
| **ABSTRACT** | | | |
| Structured summary | 2 | Provide a structured summary that includes (as applicable): background, objectives, eligibility criteria, sources of evidence, charting methods, results, and conclusions that relate to the review questions and objectives. | Abstract |
| **INTRODUCTION** | | | |
| Rationale | 3 | Describe the rationale for the review in the context of what is already known. Explain why the review questions/objectives lend themselves to a scoping review approach. | 1,2 |
| Objectives | 4 | Provide an explicit statement of the questions and objectives being addressed with reference to their key elements (e.g., population or participants, concepts, and context) or other relevant key elements used to conceptualize the review questions and/or objectives. | 3 |
| **METHODS** | | | |
| Protocol and registration | 5 | Indicate whether a review protocol exists; state if and where it can be accessed (e.g., a Web address); and if available, provide registration information, including the registration number. | 5 |
| Eligibility criteria | 6 | Specify characteristics of the sources of evidence used as eligibility criteria (e.g., years considered, language, and publication status), and provide a rationale. | 4,5 |
| Information sources* | 7 | Describe all information sources in the search (e.g., databases with dates of coverage and contact with authors to identify additional sources), as well as the date the most recent search was executed. | 5 |
| Search | 8 | Present the full electronic search strategy for at least 1 database, including any limits used, such that it could be repeated. | 30-37 |
| Selection of sources of evidence† | 9 | State the process for selecting sources of evidence (i.e., screening and eligibility) included in the scoping review. | 5,6 |
| Data charting process‡ | 10 | Describe the methods of charting data from the included sources of evidence (e.g., calibrated forms or forms that have been tested by the team before their use, and whether data charting was done independently or in duplicate) and any processes for obtaining and confirming data from investigators. | 8 |
| Data items | 11 | List and define all variables for which data were sought and any assumptions and simplifications made. | NA |
| Critical appraisal of individual sources of evidence§ | 12 | If done, provide a rationale for conducting a critical appraisal of included sources of evidence; describe the methods used and how this information was used in any data synthesis (if appropriate). | NA |
| Synthesis of results | 13 | Describe the methods of handling and summarizing the data that were charted. | 8 |
| **RESULTS** | | | |
| Selection of sources of evidence | 14 | Give numbers of sources of evidence screened, assessed for eligibility, and included in the review, with reasons for exclusions at each stage, ideally using a flow diagram. | 7 |
| Characteristics of sources of evidence | 15 | For each source of evidence, present characteristics for which data were charted and provide the citations. | 44-48 |
| Critical appraisal within sources of evidence | 16 | If done, present data on critical appraisal of included sources of evidence (see item 12). | NA |
| Results of individual sources of evidence | 17 | For each included source of evidence, present the relevant data that were charted that relate to the review questions and objectives. | 9-15 |
| Synthesis of results | 18 | Summarize and/or present the charting results as they relate to the review questions and objectives. | 9-15 |
| **DISCUSSION** | | | |
| Summary of evidence | 19 | Summarize the main results (including an overview of concepts, themes, and types of evidence available), link to the review questions and objectives, and consider the relevance to key groups. | 16 |
| Limitations | 20 | Discuss the limitations of the scoping review process. | 18 |
| Conclusions | 21 | Provide a general interpretation of the results with respect to the review questions and objectives, as well as potential implications and/or next steps. | 19-20 |
| **FUNDING** | | | |
| Funding | 22 | Describe sources of funding for the included sources of evidence, as well as sources of funding for the scoping review. Describe the role of the funders of the scoping review. | Title page |

JBI = Joanna Briggs Institute; PRISMA-ScR = Preferred Reporting Items for Systematic reviews and Meta-Analyses extension for Scoping Reviews.

* Where *sources of evidence* (see second footnote) are compiled from, such as bibliographic databases, social media platforms, and Web sites.

† A more inclusive/heterogeneous term used to account for the different types of evidence or data sources (e.g., quantitative and/or qualitative research, expert opinion, and policy documents) that may be eligible in a scoping review as opposed to only studies. This is not to be confused with *information sources* (see first footnote).

‡ The frameworks by Arksey and O’Malley (6) and Levac and colleagues (7) and the JBI guidance (4, 5) refer to the process of data extraction in a scoping review as data charting*.*

§ The process of systematically examining research evidence to assess its validity, results, and relevance before using it to inform a decision. This term is used for items 12 and 19 instead of "risk of bias" (which is more applicable to systematic reviews of interventions) to include and acknowledge the various sources of evidence that may be used in a scoping review (e.g., quantitative and/or qualitative research, expert opinion, and policy document).

*From:* Tricco AC, Lillie E, Zarin W, O'Brien KK, Colquhoun H, Levac D, et al. PRISMA Extension for Scoping Reviews (PRISMAScR): Checklist and Explanation. Ann Intern Med. 2018;169:467–473. [doi: 10.7326/M18-0850](http://annals.org/aim/fullarticle/2700389/prisma-extension-scoping-reviews-prisma-scr-checklist-explanation).
